# Antiepileptic Effects of Protein-Rich Extract from *Bombyx batryticatus* on Mice and Its Protective Effects against H_2_O_2_-Induced Oxidative Damage in PC12 Cells via Regulating PI3K/Akt Signaling Pathways

**DOI:** 10.1155/2019/7897584

**Published:** 2019-05-06

**Authors:** Meibian Hu, Yujie Liu, Liying He, Xing Yuan, Wei Peng, Chunjie Wu

**Affiliations:** ^1^School of Pharmacy, Chengdu University of Traditional Chinese Medicine, Chengdu 611137, China; ^2^School of Pharmacy, Chengdu Medical College, Chengdu 610500, China; ^3^School of Medicine and Life Sciences, Chengdu University of Traditional Chinese Medicine, Chengdu 611137, China

## Abstract

*Bombyx batryticatus* is a known traditional Chinese medicine (TCM) utilized to treat convulsions, epilepsy, cough, asthma, headaches, and purpura in China for thousands of years. This study is aimed at investigating the antiepileptic effects of protein-rich extracts from *Bombyx batryticatus* (BBPs) on seizure in mice and exploring the protective effects of BBPs against H_2_O_2_-induced oxidative stress in PC12 cells and their underlying mechanisms. Maximal electroshock-induced seizure (MES) and pentylenetetrazole- (PTZ-) induced seizure in mice and the histological analysis were carried out to evaluate the antiepileptic effects of BBPs. The cell viability of PC12 cells stimulated by H_2_O_2_ was determined by MTT assay. The apoptosis and ROS levels of H_2_O_2_-stimulated PC12 cells were determined by flow cytometry analysis. Furthermore, the levels of malondialdehyde (MDA), superoxide dismutase (SOD), lactate dehydrogenase (LDH), and glutathione (GSH) in PC12 cells were assayed by ELISA and expressions of caspase-3, caspase-9, Bax, Bcl-2, PI3K, Akt, and p-Akt were evaluated by Western blotting and quantitative real-time polymerase chain reaction (RT-qPCR) assays. The results revealed that BBPs exerted significant antiepileptic effects on mice. In addition, BBPs increased the cell viability of H_2_O_2_-stimulated PC12 cells and reduced apoptotic cells and ROS levels in H_2_O_2_-stimulated PC12 cells. By BBPs treatments, the levels of MDA and LDH were reduced and the levels of SOD and GSH-Px were increased in H_2_O_2_-stimulated PC12 cells. Moreover, BBPs upregulated the expressions of PI3K, Akt, p-Akt, and Bcl-2, whereas they downregulated the expressions of caspase-9, caspase-3, and Bax in H_2_O_2_-stimulated PC12 cells. These findings suggested that BBPs possessed potential antiepileptic effects on MES and PTZ-induced seizure in mice and protective effects on H_2_O_2_-induced oxidative stress in PC12 cells by exerting antioxidative and antiapoptotic effects via PI3K/Akt signaling pathways.

## 1. Introduction

Epilepsy, one of the most common and serious neurological disorders, could cause serious physical, psychological, social, and economic consequences [1, 2]. It is reported that epilepsy affects at least 50 million people worldwide and the median prevalence of lifetime epilepsy in developed countries and developing countries are 5.8 per 1000 and 10.3 per 1000, respectively [3]. Epilepsy is a complex disorder which may be caused from varied underlying brain pathologies, including neurodevelopmental disorders in the young, and tumors, stroke, and neurodegenerative diseases in adults [4]. Numerous neuropharmacological researches have demonstrated that development of epilepsy is closely related to neurotransmitters, ion channels, synaptic connections, glial cells, etc. [5]. In particular, oxidative stress is considered as a predominant mechanism for the pathogenesis of epilepsy [6] and several studies have revealed an increase in mitochondrial oxidative and nitrosative stress (O&NS) and subsequent cell damage after persistent seizures [7–9].

*Bombyx batryticatus* (BB), called *Jangcan* in Chinese, is the dried larva of *Bombyx mori* L. (silkworm of 4-5 instars) infected by *Beauveria bassiana* (Bals.) Vuill [10]. As a known traditional Chinese medicine (TCM), BB has been used in China for thousands of years based on its reliable therapeutic effects and it is also widely used in folk medicine of Korea and Japan [11]. BB has been utilized to treat convulsions, epilepsy, cough, asthma, headaches, and purpura in traditional Chinese medicine systems etc. [11–13]. Treatments of convulsions and epilepsy are the main traditional applications of BB, and a large number of researches have shown that extracts/compounds isolated from BB possess significant anticonvulsant and antiepileptic effects on different animal models [14–16]. However, current investigations of BB mainly focus on its small molecule compounds but rarely investigate its macromolecular compounds. Interestingly, our previous study has indicated that the anticonvulsant effect of BB powder is obviously stronger than that of BB decoction on mice [17]. As an animal Chinese medicine, the main chemical constituents in BB are proteins. Thus, it is quite essential to evaluate the anticonvulsant and antiepileptic effects of proteins in BB in order to clear whether proteins are the main active substances corresponding to the anticonvulsant or antiepileptic effects of BB or not. In previous studies, it has been shown that proteins isolated from TCMs possess various bioactivities, such as antitumor, antioxidant, immunomodulatory, and hypoglycemic effects [18]. However, to the best of our knowledge, there is no systematical research on proteins from BB [19]. Therefore, to explore the antiepileptic substance basis of BB, the antiepileptic effects of protein-rich extracts from BB (BBPs) on MES and PTZ-induced seizure in mice were carried out in the present study. Protective effects of BBPs against H_2_O_2_-induced oxidative damage in PC12 cells and their underlying mechanisms were also studied.

## 2. Materials and Methods

### 2.1. Materials and Chemicals

BB medicinal materials were purchased from Chengdu *Min-Jiang-Yuan* Pharmaceutical Co. Ltd. (Chengdu, China) and were identified by Prof. Chun-Jie Wu (School of Pharmacy, Chengdu University of Traditional Chinese Medicine, Chengdu, China). A voucher specimen (Y170628) was deposited in the School of Pharmacy, Chengdu University of Traditional Chinese Medicine (Chengdu, China). Pentylenetetrazole (PTZ), DILANTIN, diazepam, and H_2_O_2_ were purchased from Sigma-Aldrich Co. (St. Louis, MO, USA). The DMEM media, fetal bovine serum (FBS), and horse serum were obtained from Invitrogen (Carlsbad, California, CA, USA). The 3-(4,5-dimethylthiazol-2-yl)-2,5-diphenyltetrazolium bromide (MTT) was purchased from Beyotime Institute of Biotechnology (Shanghai, China). Malondialdehyde (MDA), superoxide dismutase (SOD), lactate dehydrogenase (LDH), and glutathione (GSH-Px) ELISA kits were products of Shanghai ZhuoCai Biotechnology Co. Ltd. (Shanghai, China). Caspase-3, caspase-9, Bax, Bcl-2, PI3K, Akt, p-Akt, and β-actin antibodies were purchased from Abcam (Cambridge, USA). Annexin V/FITC kit and reactive oxygen species (ROS) testing kit were purchased from the BD Biosciences (Shanghai, China). Bicinchoninic acid (BCA) protein assay reagent and horseradish peroxidase- (HPR-) conjugated secondary antibody were purchased from Beyotime Institute of Biotechnology (Shanghai, China). RNA TRIzol Reagent was purchased from Servicebio Company (Wuhan, China). The RevertAid First Strand cDNA Synthesis Kit was purchased from Thermo Fisher (MO, USA). All other reagents used in the experiments were of analytical grade.

### 2.2. Protein Extraction

BB was powdered and defatted with petroleum ether (1 : 5, *w*/*v*) as the previously reported [20]. BBPs were extracted according to the method reported by Chen et al. [21]. Briefly, defatted BB was mixed with phosphate buffer (pH 8.0, 30 mM) at a ratio of 1 : 3 (*w*/*v*), extracted on ice for 1 h with ultrasonic extraction and subsequently centrifuged at 5000 rpm for 30 min. The soluble proteins in the supernatant was fractional precipitated by saturated (80%) ammonium sulfate ((NH4)_2_SO_4_) at 4°C overnight and then centrifuged at 5000 rpm for 30 min. The obtained precipitates were redissolved in PBS and dialyzed at 4°C for 24 h against distilled water using 30 kDa dialysis membranes and lyophilized. The yield of BBPs is 2.16% and the protein content is 71.98%.

### 2.3. Protein Patterns by SDS-PAGE

SDS-PAGE analysis was carried out as previously reported by Laemmli with some modifications [22]. A vertical slab gel of 1.5 mm thickness was used, and protein was separated by SDS-PAGE with 12% acrylamide separating gel and 4% stacking gel (Bio-Rad Laboratories, Mini-PROTEAN 3 Cell). SDS-PAGE patterns were analyzed using Image Lab software (Bio-Rad, 6.0.1, USA) to obtain the molecular weight and relative content of proteins (relative to M-9). Prestained protein markers (10-180 kDa, Thermo Fisher, Waltham, Ma, USA) were used as standard.

### 2.4. Animals

Male Kunming mice (25-30 g) were purchased from Chengdu *Dashuo* Experimental Animal Co. Ltd. (Chengdu, China), maintained at 22 ± 2°C and 12 h light-dark cycle with free access to food and water. Mice were allowed to adapt to the laboratory for 3 days before experiments. All animal experimental protocols were performed in accordance with international regulations on the care and use of laboratory animals and were approved by the Animal Care and Use Committee of Chengdu University of Traditional Chinese Medicine (Chengdu, China).

### 2.5. Antiepileptic Effects of BBPs on Mice

#### 2.5.1. Maximal Electroshock-Induced Seizure (MES) Test

MES in mice were induced by an intensity of 50 mA at 60 Hz alternating current via JTC-1 seizure and pain experiment alternating current stimulator. The current was applied for 0.3 s via ear clip electrodes, coated with an electrolyte solution. A total of 100 mice were divided into 5 groups (*n* = 20): model, positive, and three BBP-treated groups (0.75, 1.5, and 3 g/kg). BBP-treated mice were orally administered for 7 days pretreatment (0.75, 1.5, and 3 g/kg). Mice in model and positive groups were administered orally with normal saline (10 mL/kg). On the 7th day, MES tests of all mice were performed after 1 h treatment with saline (model group), BBPs (BBP-treated groups), or DILANTIN (positive group, 20 mg/kg). The tetanic convulsion of hind limbs of mice was used as the index of seizure, and the seizure rates were calculated.

#### 2.5.2. PTZ-Induced Seizure Test

Sixty male mice were divided into 6 groups (*n* = 10): normal, model, positive, and three BBP-treated groups (0.75, 1.5, and 3 g/kg). BBP-treated mice were orally administered for 7 days pretreatment (0.75, 1.5, and 3 g/kg). During 7 days pretreatment, mice in the normal, model, and positive groups were administered orally with normal saline (10 mL/kg). On the 7th day, seizure tests induced by PTZ (85 mg/kg; i.p.) of all mice except the normal group were performed after 30 min treatment with saline (model group), BBPs (BBP-treated groups), or diazepam (positive group; 1 mg/kg; i.p.). The latency to the initiation of the first seizure and death (anticonvulsant response) was evaluated during 30 min [23].

#### 2.5.3. Histological Analysis

After PTZ-induced seizures, mice were euthanized and the entire brain was removed for histological analysis according to routine histological methods (embedded in paraffin and blocks cut into sections of 5 *μ*m thickness). Brain tissues were sectioned at the hippocampal coronal plane. Sections were stained with hematoxylin and eosin (HE) and toluidine blue. Hippocampal cornu ammonis 1 (CA1) and cornu ammonis 3 (CA3) areas were photographed using a BA200 digital microscope (McAudi, Xiamen, China). Additionally, the total number of dark neurons was analyzed (10 sections/animal) in slides stained with toluidine blue according to the previous report [24]. The number of dark neurons in the CA1 and CA3 areas was analyzed.

### 2.6. Protective Effects of BBPs on H_2_O_2_-Stimulated PC12 Cells In Vitro

#### 2.6.1. Cell Culture

Rat pheochromocytoma-derived cell line PC12 cells were obtained from Wuhan Pu-nuo-sai Life Technology Co. Ltd. (Wuhan, China) and maintained in Dulbecco's modified Eagle's medium (DMEM) supplemented with 10% horse serum, 5% fetal bovine serum (FBS), and 1% penicillin/streptomycin. Cells were incubated at 37°C in a 5% CO_2_ atmosphere [25].

#### 2.6.2. Cell Viability Assay

The cell viability was evaluated by the 3-(4,5-dimethylthiazol-2-yl)-2,5-diphenyltetrazolium bromide (MTT) assay. Cells (3000 cells/well) were plated in 96-well plates for growth of 24 h. To evaluate the effect of BBPs on cell viability, BBPs at the final concentrations of 50, 100, 200, 400, and 800 *μ*g/mL were added and cultured at 37°C for 24 h. The medium was removed, and MTT solution was added to the culture medium and incubated at 37°C for 4 h. Then optical density (OD) values were measured at 490 nm using a Multlskan Mk3 microplate reader (Thermo Fisher, Waltham, MA, USA). The experiment was repeated thrice. Cell viability was expressed as a percentage OD value of the normal (untreated) cells.

Additionally, to evaluate the effect of BBPs on the H_2_O_2_-stimulated PC12 cell viabilities, cells were presented with BBPs at concentrations of 50, 100, 200, 400, and 800 *μ*g/mL for 24 h at 37°C and subsequently subjected to H_2_O_2_ at the concentration of 300 *μ*mol/L for 4 h. MTT assay was performed as the above method.

#### 2.6.3. Apoptosis and ROS Assays by Flow Cytometry Analysis

Cells were plated in the 24-well plates for growth of 24 h. Then BBPs (final concentrations of 200, 400, and 800 *μ*g/mL) were added to the cells and cultured for 24 h at 37°C. Next, H_2_O_2_ (final concentration of 300 *μ*mol/L) was added and cultured for another 4 h. Cells (5 × 10^4^) were harvested and washed using PBS and stained by the Annexin V/FITC kit. Cell apoptosis was detected by the flow cytometry (FCM) assay on a FACSCalibur flow cytometer (BD Biosciences, USA). In addition, the DCFH-DA ROS kit was used to determine the intracellular ROS level by FCM assay. All experiments were repeated thrice.

#### 2.6.4. Determination of MDA, SOD, LDH, and GSH-Px Contents in PC12 Cells

Cells were inoculated into the 24-well plates for 24 h. BBPs at the final concentrations of 200, 400, and 800 *μ*g/mL were added and cultured for another 24 h. Next, H_2_O_2_ at the final concentration of 300 *μ*mol/L was added and cultured for 4 h. Cells were harvested, and contents of MDA, SOD, LDH, and GSH-Px were determined by ELSA kits following the manufacturer's instruction using a Multlskan Mk3 Microplate Reader (Thermo Fisher, Waltham, MA, USA).

#### 2.6.5. Western Blot Assay

Total proteins of the PC12 cells were extracted, and protein concentration was determined using BCA protein assay reagent. Total proteins (35 *μ*g) were separated by 12% SDS-PAGE and then transferred onto a PVDF membrane. After that, the PVDF membrane was incubated with primary antibodies of caspase-3, caspase-9, Bax, Bcl-2, PI3K, Akt, and p-Akt at 4°C overnight. The membrane was washed and further incubated with HPR-conjugated secondary antibodies at room temperature for 1 h. Protein bands were detected by chemiluminescence, and *β*-actin was used as the internal reference.

#### 2.6.6. Quantitative Real-Time Polymerase Chain Reaction (RT-qPCR) Assay

Total RNA of the PC12 cells was extracted according to the manufacturer's instruction, and their purity and concentration were determined by their absorbance at 260 and 280 nm. Then 2 *μ*g RNA was reversely transcribed into cDNA using the RevertAid First Strand cDNA Synthesis Kit. RT-qPCR was performed using an ABI StepOnePlus System (Applied Biosystems, CA, USA). The reaction process for the RT-qPCR was as follows: 95°C for 30 s, followed by cycles of 95°C for 5 s and 55°C for 30 s and then 72°C for 30 s. The gene expressions of caspase-3, caspase-9, Bax, Bcl-2, PI3K, Akt, and p-Akt were normalized to *β*-actin and analyzed by using 2^−△△CT^ method.

### 2.7. Statistical Analysis

Data are presented as mean ± standard deviations (SD). Statistical comparisons except the seizure rate were made by Student's *t*-test or one-way analysis of variance (ANOVA) using GraphPad Prism 5 software (GraphPad Software Inc., La Jolla, CA). Comparisons of the seizure rate were made by chi-square test using SPSS software (version 20.0, USA). *P* < 0.05 was set as the significant level.

## 3. Results

### 3.1. Protein Patterns by SDS-PAGE

**Figure 1:**
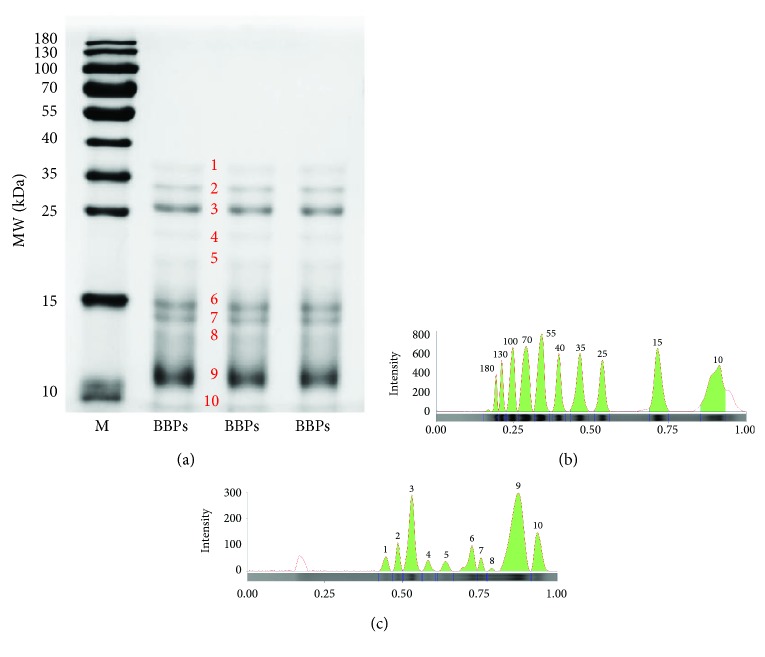
SDS-PAGE analysis of BBPs. (a) SDS-PAGE patterns of BBPs. (b) Electrophoresis lane of the marker. (c) Electrophoresis lane of BBPs. M: standard marker; BBPs: protein extracts in *Bombyx batryticatus.* SDS-PAGE patterns were analyzed using Image Lab software 6.0.1.

The SDS-PAGE information of BBPs was presented in Figure 1 and Table 1. It is shown that the molecular weight (MW) of proteins composed of BBPs was below 40 kDa. It can be observed in Table 2; 10 proteins in BBPs were detected using Image Lab software, including the MW of 10.00, 10.66, 12.74, 13.74, 14.62, 18.43, 21.63, 25.28, 31.20, and 36.00 kDa, and their protein contents (relative to M-9, 15 kDa) were 0.21, 0.75, 0.01, 0.04, 0.12, 0.04, 0.04, 0.34, 0.08, and 0.06, respectively. Therefore, the relative contents of proteins 3, 9, and 10 were higher than those of other proteins in BBPs.

### 3.2. Antiepileptic Effects of BBPs on MES and PTZ-Induced Seizure in Mice

The antiepileptic effects of BBPs were shown in Tables 2 and 3. The results of the MES test showed that the seizure rate of mice in the model, positive, and BBP-treated groups were 100%, 0, 85%, 75%, and 60%, respectively. And it also showed that the seizure rates of the BBP-treated groups (1.5 and 3 g/kg; *P* < 0.05) and the positive group (*P* < 0.05) were significantly lower than that of the model group. As shown in Table 3, the latency of PTZ-induced seizures and that of death of the model mice were 79.00 ± 11.58 s and 171.40 ± 30.96 s, respectively. BBPs at doses of 1.5 and 3 g/kg (*P* < 0.05) obviously increased the latency of PTZ-induced seizures and death in mice compared with the model group. In addition, 20% of the animals in the BBP-treated (800 mg/kg) group survived, while 70% of the animals in the diazepam-treated group survived.

**Figure 2:**
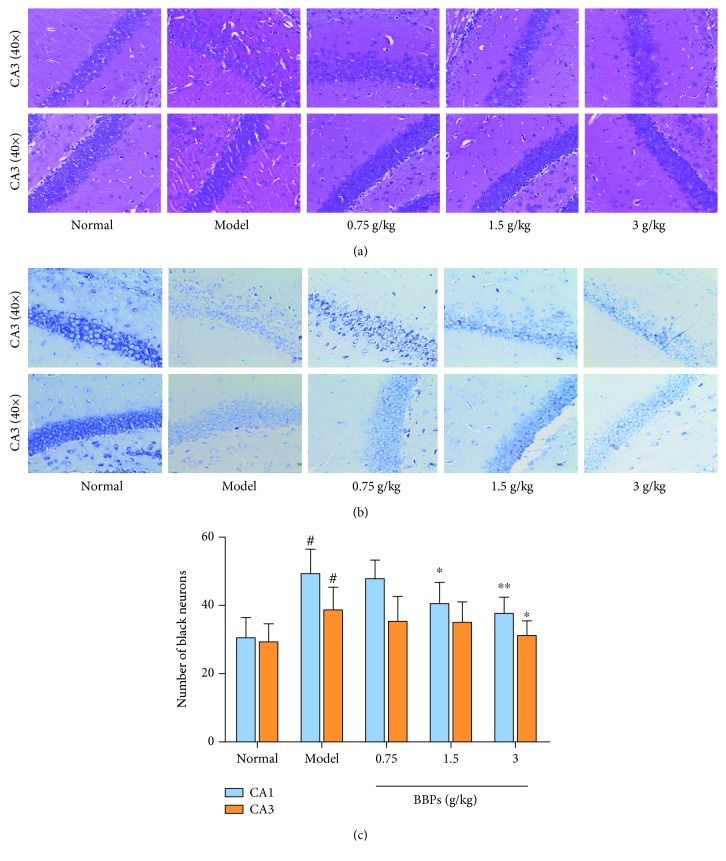
Histological analysis. (a) Hematoxylin and eosin stain (40x). (b) Toluidine blue stain (40x). (c) Number of black neurons in the hippocampal CA1 and CA3 areas stained by toluidine blue. BBPs: protein extracts in *Bombyx batryticatus.* The values represent mean ± SD (*n* = 6). ^#^*P* < 0.05 and ^##^*P* < 0.01 vs. the normal group; ^∗^*P* < 0.05 and ^∗∗^*P* < 0.01 vs. the model group.

Results of histological analysis were presented in Figure 2, including H&E stain (Figure 2(a)) and toluidine blue stain (Figure 2(b)). It can be seen that the morphology, number, and distribution of hippocampal cells were normal and apoptotic cells were not found in normal mice. However, hippocampal cells of the model mice had some significant pathological changes compared with those of the normal mice especially in the CA1 area, such as cell irregular morphology, cell number decreasing, and nuclear pyknosis. Interestingly, different doses of BBPs significantly improved the pathological changes of hippocampal cells, relative to the model mice. In addition, the histological evaluation of the hippocampus stained by toluidine blue demonstrated that the number of dark neurons in the CA1 area significantly increased in the model group (49 ± 7, *P* < 0.05) compared with that in the normal group (31 ± 6). BBPs at doses of 1.5 (40 ± 6, *P* < 0.05) and 3 g/kg (38 ± 5, *P* < 0.05) significantly reduced the number of dark neurons in the CA1 area, compared with that in the model mice. However, BBPs had no significant effects on the CA3 area of the hippocampus in mice.

### 3.3. Protective Effects of BBPs on the Cell Viability of H_2_O_2_-Stimulated PC12 Cells

**Figure 3:**
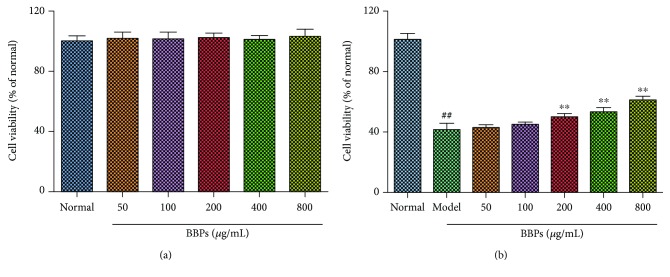
Protective effects of BBPs on the cell viability of H_2_O_2_-stimulated PC12 cells. (a) Effects of BBPs on cell viability of PC12 cell. PC12 cells were treated with BBPs at concentrations ranging from 0 to 800 *μ*g/mL for 24 h. (b) Effects of BBPs on cell viability of H_2_O_2_-induced PC12 cells. PC12 cells were treated with BBPs at concentrations ranging from 0 to 800 *μ*g/mL for 24 h, subsequently subjected to H_2_O_2_ at the concentration of 300 *μ*mol/L for 4 h. The cell viability was determined by MTT assay. BBPs: protein extracts in *Bombyx batryticatus.* The values represent mean ± SD (*n* = 6). ^##^*P* < 0.01 vs. the normal group; ^∗∗^*P* < 0.01 vs. the model group.

The effects of BBPs on the cell viability of PC12 cells and H_2_O_2_-stimulated PC12 cells were evaluated by MTT assay. As shown in Figure 3(a), BBPs did not show any toxicity of up to the concentration of 800 *μ*g/mL in normal cells. The cell viability (Figure 3(b)) was significantly reduced by treatment with H_2_O_2_ (*P* < 0.01). Furthermore, BBPs increased the cell viability of H_2_O_2_-stimulated PC12 cells from the concentrations of 200 to 800 *μ*g/mL in a dose-dependent manner, relative to the model cells (*P* < 0.01). Consequently, the results indicated that BBPs had a potential protective effect against the H_2_O_2_-induced injury in PC12 cells.

### 3.4. Effects of BBPs on H_2_O_2_-Induced Apoptosis of PC12 Cells

**Figure 4:**
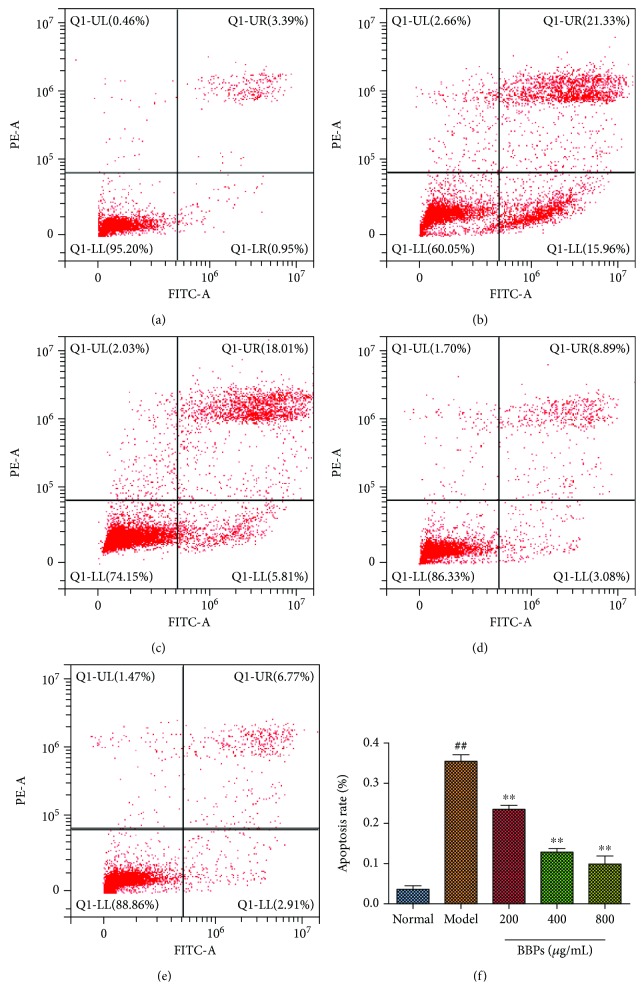
Effects of BBPs on H_2_O_2_-stimulated apoptosis of PC12 cells. (a) Normal group. (b) Model group. (c) 200 *μ*g/mL of BBP group. (d) 400 *μ*g/mL of BBP group. (e) 800 *μ*g/mL of BBP group. (f) Apoptosis rate of different groups. PC12 cells were treated with BBPs at concentrations of 200, 400, and 800 *μ*g/mL for 24 h, subsequently subjected to H_2_O_2_ at the concentration of 300 *μ*mol/L for 4 h. Cell apoptosis was detected by the flow cytometry assay. BBPs: protein extracts in *Bombyx batryticatus.* The values represent mean ± SD (*n* = 3). ^##^*P* < 0.01 vs. the normal group; ^∗∗^*P* < 0.01 vs. the model group.

To evaluate the effects of BBPs on H_2_O_2_-induced apoptosis of PC12 cells, the FCM assay with Annexin V-FITC/PI staining was carried out. As shown in Figure 4, after treatment with H_2_O_2_, the percentage of apoptotic cells sharply increased compared with that of the normal PC12 cells (*P* < 0.01). However, our results also showed that BBPs (200, 400, and 800 *μ*g/mL) reduced the percentage of apoptotic cells in dose-dependent manners (*P* < 0.01, *P* < 0.01, and *P* < 0.01), relative to the model PC12 cells.

### 3.5. Effects of BBPs on ROS Levels of H_2_O_2_-Stimulated PC12 Cells

**Figure 5:**
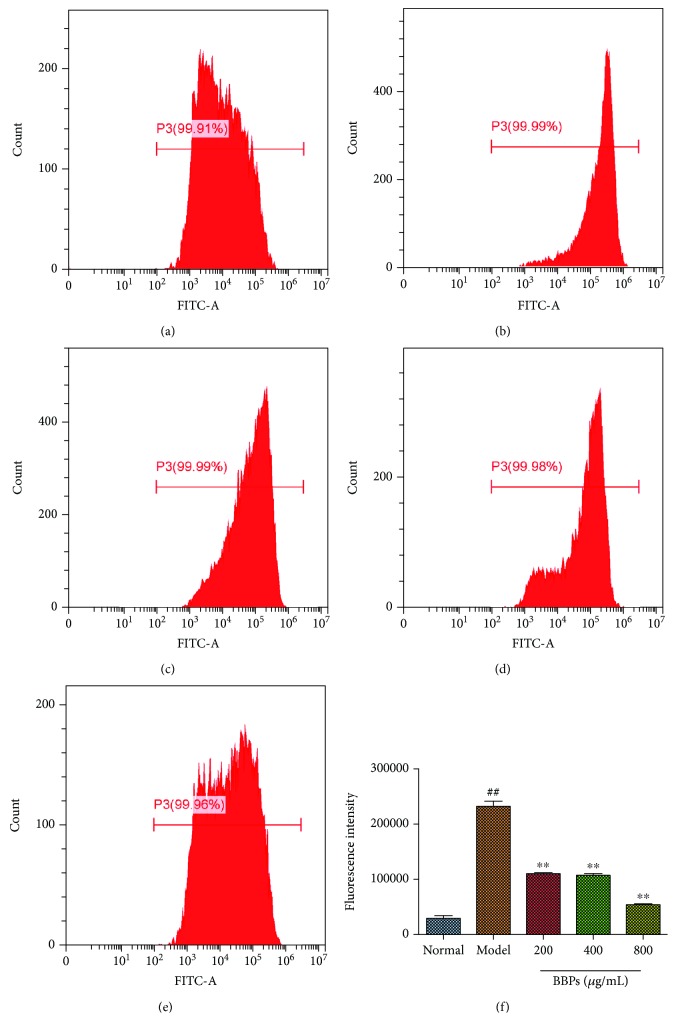
Effects of BBPs on ROS levels of H_2_O_2_-stimulated PC12 cells. (a) Normal group. (b) Model group. (c) 200 *μ*g/mL of BBP group. (d) 400 *μ*g/mL of BBP group. (e) 800 *μ*g/mL of BBP group. (f) Fluorescence intensity of different groups. PC12 cells were treated with BBPs at concentrations of 200, 400, and 800 *μ*g/mL for 24 h, subsequently subjected to H_2_O_2_ at the concentration of 300 *μ*mol/L for 4 h. The intracellular ROS level by the flow cytometry (FCM) assay. BBPs: protein extracts in *Bombyx batryticatus.* The values represent mean ± SD (*n* = 3). ^##^*P* < 0.01 vs. the normal group; ^∗^*P* < 0.05 and ^∗∗^*P* < 0.01 vs. the model group.

To evaluate the effects of BBPs against oxidative stress induced by H_2_O_2_ in PC12 cells, the ROS levels were measured by FCM assay with DCFH-DA staining. As shown in Figure 5, the ROS level of the model cells significantly increased compared with that of the normal cells (*P* < 0.01). However, our results also demonstrated that BBPs (200, 400, and 800 *μ*g/mL) could dose-dependently reduce the ROS level in PC12 cells stimulated by H_2_O_2_ (*P* < 0.01, *P* < 0.01, and *P* < 0.01), compared with that in the model cells.

### 3.6. Effects of BBPs on MDA, SOD, LDH, and GSH-Px in H_2_O_2_-Stimulated PC12 Cells

**Figure 6:**
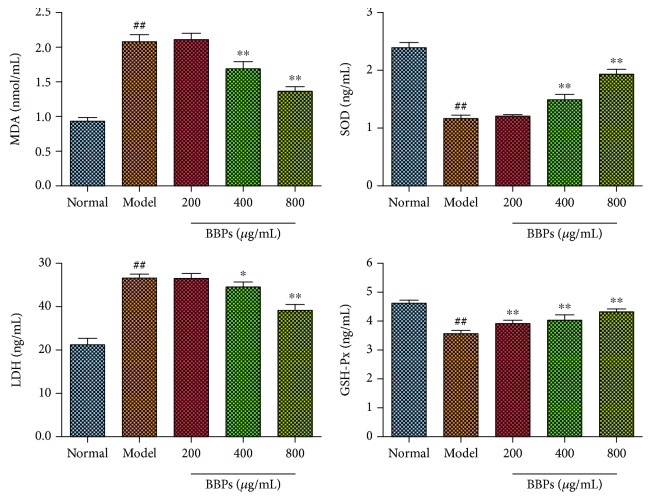
Effects of BBPs on MDA, SOD, LDH, and GSH-Px in H_2_O_2_-stimulated PC12 cells. The levels of MDA, SOD, LDH, and GSH-Px were determined by the corresponding ELISA kits. PC12 cells were treated with BBPs at concentrations of 200, 400, and 800 *μ*g/mL for 24 h, subsequently subjected to H_2_O_2_ at the concentration of 300 *μ*mol/L for 4 h. BBPs: protein extracts in *Bombyx batryticatus.* The values represent mean ± SD (*n* = 6). ^##^*P* < 0.01 vs. the normal group; ^∗^*P* < 0.05 and ^∗∗^*P* < 0.01 vs. the model group.

We also investigated the effects of BBPs on the levels of MDA, SOD, LDH, and GSH-Px in H_2_O_2_-stimulated PC12 cells. As shown in Figure 6, the levels of SOD and GSH-Px were significantly decreased (*P* < 0.01, *P* < 0.01), whereas the MDA and LDH contents were obviously increased (*P* < 0.01) in model cells compared with the normal cells. Interestingly, BBPs (400 and 800 *μ*g/mL) significantly reduced MDA (*P* < 0.01, *P* < 0.01) and LDH (*P* < 0.05, *P* < 0.01) contents compared with the model cells. The SOD level was obviously increased in BBP-treated cells (400 and 800 *μ*g/mL; *P* < 0.01, *P* < 0.01), compared with the model cells. The GSH-Px contents in all BBP-treated cells (*P* < 0.01, *P* < 0.01, and *P* < 0.01) were increased in a dose-dependent manner, compared with those in the model cells.

### 3.7. Effects of BBPs on mRNA Expressions of Caspase-3, Caspase-9, Bax, Bcl-2, PI3K, Akt, and p-Akt in H_2_O_2_-Stimulated PC12 Cells

**Figure 7:**
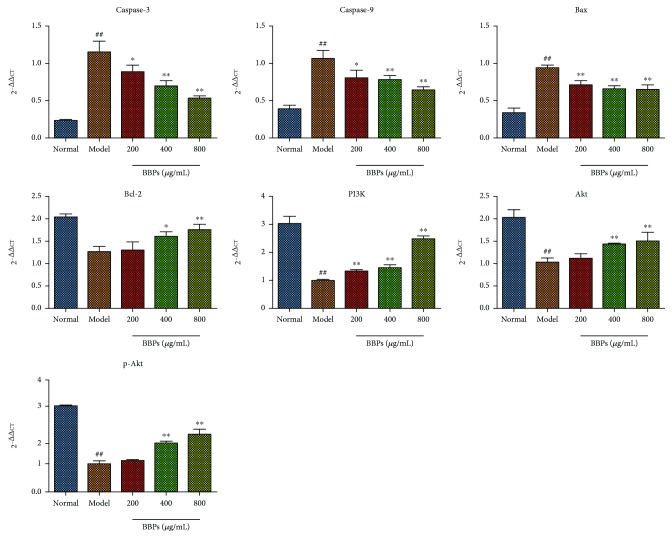
Effects of BBPs on mRNA expressions of caspase-3, caspase-9, Bax, Bcl-2, PI3K, Akt, and p-Akt in H_2_O_2_-stimulated PC12 cells. PC12 cells were treated with BBPs at concentrations of 200, 400, and 800 *μ*g/mL for 24 h, subsequently subjected to H_2_O_2_ at the concentration of 300 *μ*mol/L for 4 h. Caspase-3, caspase-9, Bax, Bcl-2, PI3K, Akt, and p-Akt mRNAs were detected by RT-qPCR, whereas *β*-actin was detected as the control. BBPs: protein extracts in *Bombyx batryticatus.* The values represent mean ± SD (*n* = 3). ^##^*P* < 0.01 vs. the normal group; ^∗^*P* < 0.05 and ^∗∗^*P* < 0.01 vs. the model group.

As shown in Figure 7, the mRNA expressions of caspase-3, caspase-9, and Bax in the model cells were obviously upregulated, whereas Bcl-2 expression was downregulated, compared with those in the normal cells (*P* < 0.01). Compared with the model cells, the caspase-3, caspase-9, and Bax mRNA expressions were significantly decreased in all BBP-treated (200, 400, and 800 *μ*g/mL) cells, while the Bcl-2 (*P* < 0.05) expression was increased by treatment with BBPs of 400 and 800 *μ*g/mL. Additionally, mRNA expressions of PI3K, Akt, and p-Akt in model cells were obviously downregulated compared with the those in normal cells. BBP (400 and 800 *μ*g/mL) treatment significantly increased these mRNA (PI3K, Akt, and p-Akt) expressions compared with the model cells.

### 3.8. Effects of BBPs on Protein Expressions of Caspase-3, Caspase-9, Bax, Bcl-2, PI3K, Akt, and p-Akt in H_2_O_2_-Stimulated PC12 Cells

**Figure 8:**
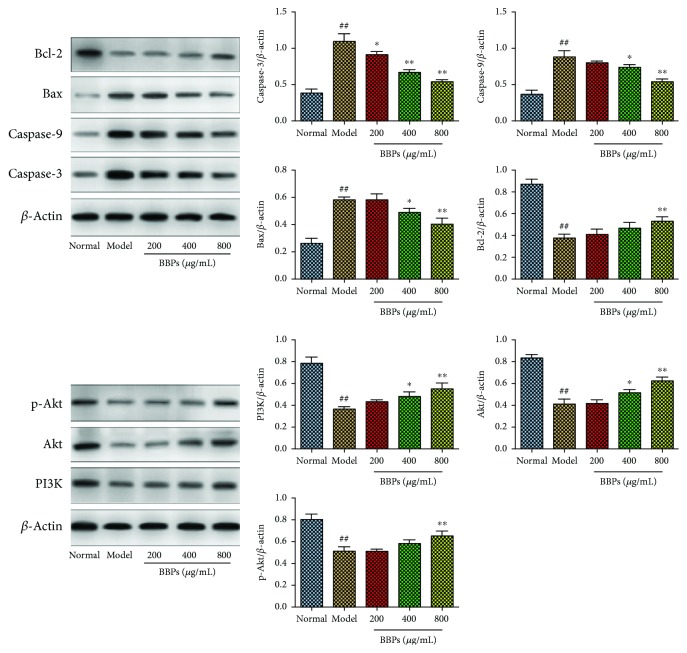
Effects of BBPs on protein expressions of caspase-3, caspase-9, Bax, Bcl-2, PI3K, Akt, and p-Akt in H_2_O_2_-stimulated PC12 cells. PC12 cells were treated with BBPs at concentrations of 200, 400, and 800 *μ*g/mL for 24 h, subsequently subjected to H_2_O_2_ at the concentration of 300 *μ*mol/L for 4 h. Caspase-3, caspase-9, Bax, Bcl-2, PI3K, Akt, and p-Akt proteins were detected by Western blotting, whereas *β*-actin was detected as the control. BBPs: protein extracts in *Bombyx batryticatus.* The values represent mean ± SD (*n* = 3). ^##^*P* < 0.01 vs. the normal group; ^∗^*P* < 0.05 and ^∗∗^*P* < 0.01 vs. the model group.

To explore molecular mechanisms of the antiapoptotic and protective effects of BBPs on H_2_O_2_-stimulated PC12 cells, expressions of apoptosis-related and oxidative stress-related proteins were determined. As shown in Figure 8, the results indicated that after treatment with H_2_O_2_, protein expressions of caspase-3, caspase-9, and Bax in PC12 cells were significantly upregulated, whereas Bcl-2 was significantly downregulated, compared with those in the cells in the normal group (*P* < 0.01). Interestingly, BBPs at all the tested concentrations significantly decreased the caspase-3 protein expression (*P* < 0.05). BBPs significantly decreased the protein expressions of caspase-9 and Bax at the concentrations of 400 and 800 *μ*g/mL (*P* < 0.05, *P* < 0.01), and only BBPs at a concentration of 800 *μ*g/mL increased Bcl-2 (*P* < 0.01) expression in H_2_O_2_-stimulated PC12 cells, relative to the model cells. Additionally, the results showed that after the treatment with H_2_O_2_, protein expressions of PI3K, Akt, and p-Akt were significantly downregulated (*P* < 0.01), compared with those of the normal group. However, the protein expressions of PI3K and Akt were significantly increased after the treatment with BBPs at the concentrations of 400 and 800 *μ*g/mL (*P* < 0.05 and *P* < 0.01, respectively), and only BBPs at a concentration of 800 *μ*g/mL significantly increased the p-Akt protein expression (*P* < 0.01), relative to the model cells.

## 4. Discussion

Epilepsy is a brain dysfunction characterized by sudden abnormal discharges from local brain lesions [26]. Currently, animal models used to study epilepsy mainly include acute, chronic, and hereditary epilepsy models. Acute animal models include MES and PTZ-induced seizure models, which are regarded as the effective ways for the first screening of epileptic drugs [27]. The hippocampus is one of the most vulnerable brain areas for lesions resultant from excitotoxicity, especially the CA1 and CA3 regions [24]. Dark neurons were identified by neuronal shrinkage, cytoplasmic eosinophilia, nuclear pyknosis, and surrounding spongiosis, which are traditionally used to represent a typical morphological change of injured neurons [27, 28]. The present study adopted MES and PTZ-induced seizure models to explore the antiepileptic effect of BBPs extracted from *Bombyx batryticatus in vivo*. The results indicated that BBPs at doses of 1.5 and 3 g/kg possessed significant antiepileptic effects on MES and PTZ-induced seizure in mice mainly acted on the CA1 region.

It is demonstrated that many free radicals are produced in the development of central nervous system neurodegenerative diseases, such as stroke, dementias, and Parkinson's disease [29, 30]. Oxidative stress is considered as one of the predominant mechanisms in the pathogenesis of epilepsy [6]. In addition, reactive oxygen species (ROS) could cause oxidative damage of the biomacromolecules and tissues of the body and eventually lead to neuronal degeneration and necrosis [31, 32]. Hydrogen peroxide (H_2_O_2_) could easily go through the cytomembrane and form the powerful radicals, such as hydroxyl radical and singlet oxygen; therefore, H_2_O_2_ is commonly used as an inducer of oxidative-damaged cells [33]. Furthermore, PC12 cells (a rat pheochromocytoma-derived cell line) with good neuronal properties are widely used as nerve cell models to study neurological diseases, such as Alzheimer disease (AD), epilepsy, and schizophrenia [34, 35]. In our study, the H_2_O_2_-induced oxidative-damaged PC12 cells were successfully prepared and results demonstrated that BBPs could significantly alleviate the cell viability inhibition and apoptosis induced by H_2_O_2_ in PC12 cells.

Intracellular MDA, SOD, GSH, and LDH are important biomarkers to evaluate the oxidative stress level in cells or tissues. MDA is the production of peroxidation of membrane lipids induced by ROS, which could result in membrane damage and destruction, while SOD and GSH are regarded as key antioxidative enzymes in mammalian cells [36, 37]. In addition, LDH is a cytoplasmic marker enzyme, which will be quickly released after oxidative damage of cells, so LDH is a sensitive index to reflect the degree of membrane damage [38]. Therefore, MDA, SOD, GSH, and LDH contents were detected in the present study. Results showed that BBPs could significantly reduce the levels of MDA and LDH, whereas BBPs increase SOD and GSH contents in H_2_O_2_-stimulated PC12 cells. Additionally, results of the FCM assay also indicated that BBPs could obviously decrease the ROS levels in H_2_O_2_-stimulated PC12 cells. All these results indicated that BBPs could significantly reduce the oxidative damage in PC12 cells.

Recent studies have shown that the activation of the PI3K/Akt pathway is critical for neuronal survival by promoting cell survival and inhibiting apoptosis [39]. The PI3K/Akt pathway plays an antioxidant role in both central and peripheral neurons, which is also regarded as one of the cell protection mechanisms of H_2_O_2_-induced cell damage [40]. Akt, a serine/threonine kinase, is the key mediator of PI3K-initiated signaling and can promote neuronal survival by regulating expressions of caspase-9, Bcl-2, Bax, etc. [41, 42]. Phosphorylation of Akt (p-Akt) is the direct result of PI3K activation and can be used as an indicator of PI3K activation [43]. It is generally recognized that oxidative stress could induce neuronal apoptosis and caspase family proteins are the executor of cell apoptosis and caspase-9 is considered as the initiating caspase in the caspase cascade reaction [44]. Caspase-3 activated by caspase-9 is a crucial death protease, which is considered as a biomarker to identify whether cells are undergoing apoptosis [44]. In addition, Bcl-2 has been recognized for its prosurvival, antioxidant, antiapoptotic, and cytoprotective functions [45]. However, Bax can directly promote the release of cytochrome *c* into the cytoplasm and inhibit antiapoptotic Bcl-2 proteins [45]. In our study, BBPs upregulated mRNA and protein expressions of PI3K, Akt, p-Akt, and Bcl-2, whereas BBPs downregulated those of caspase 9, caspase 3 and Bax in H_2_O_2_-stimulated PC12 cells. The results above demonstrated that BBPs possessed significant antiapoptotic and protective effects on oxidative damage induced by H_2_O_2_ via regulating PI3K/Akt signaling.

In addition, according to the results of SDS-PAGE analysis, we speculated that the antiepileptic and neuroprotective effects of BBPs were possibly related to proteins with higher contents in BBPs, such as proteins 3 (25.28 kDa), 6 (14.62 kDa), 9 (10.66 kDa), and 10 (10.00 kDa) and further investigations of BBPs will be done in the future, such as their purification and separation, activities, and structure of pure protein.

## 5. Conclusions

Collectively, the present study evaluated the antiepileptic effects of BBPs on MES and PTZ-induced seizure in mice and the protective effects of BBPs against H_2_O_2_-induced injury in PC12 cells and their underlying mechanisms. Our results showed that BBPs possessed notable antiepileptic effects and exerted protective effects against oxidative damage of PC12 cells induced by H_2_O_2_ through regulating the PI3K/Akt signaling pathway. The present study provides scientific basis for *Bombyx batryticatus* of traditional usage in treating convulsions and epilepsy.

## Figures and Tables

**Table 1:** SDS-PAGE information of BBPs.

Protein band	M		BBPs	
	Molecular weight (kDa)	Relative quantification	Molecular weight (kDa)	Relative quantification
1	180.00	0.19	36.00	0.06
2	130.00	0.30	31.20	0.08
3	100.00	0.63	25.28	0.34
4	70.00	0.98	21.63	0.04
5	55.00	1.02	18.43	0.04
6	40.00	0.57	14.62	0.12
7	35.00	0.79	13.74	0.04
8	25.00	0.61	12.74	0.01
9	15.00	1.00	10.66	0.75
10	10.00	1.37	10.00	0.21

M: standard marker; BBPs: protein extracts in *Bombyx batryticatus*.

**Table 2:** Antiepileptic effect of BBPs on pentylenetetrazole- (PTZ-) induced seizure in mice (*n* = 10).

Group	Seizure latency (s)	Animals with seizure (%)	Death latency (s)	Survival animals (%)
Model	79.00 ± 11.58	100	171.40 ± 30.96	0
Positive	165.80 ± 21.35^∗^	100	1019.20 ± 125.44^∗^	80
BBPs (0.75 g/kg)	79.50 ± 15.95	100	187.80 ± 21.90	0
BBPs (1.5 g/kg)	88.80 ± 8.57^∗^	100	200.90 ± 30.61^∗^	10
BBPs (3 g/kg)	162.4 ± 22.59^∗^	100	521 ± 114.60^∗^	20

BBPs: protein extracts in *Bombyx batryticatus*; ^∗^*P* < 0.05 vs. the model.

**Table 3:** Antiepileptic effect of BBPs on maximal electroshock-induced seizure (MES) in mice (*n* = 10).

Group	N	Nc	Nn	Seizure rate (%)
Model	20	20	0	100
Positive	20	0	20	0^∗^
BBPs (0.75 g/kg)	20	17	3	85
BBPs (1.5 g/kg)	20	15	5	75^∗^
BBPs (3 g/kg)	20	12	8	60^∗^

N: number of mice; Nc: number of convulsant mice; Nn: number of nonconvulsant mice; BBPs: protein extracts in *Bombyx batryticatus*; ^∗^*P* < 0.05 vs. the model.

## Data Availability

The data used to support the findings of this study are included within the article.
